# Mesenchymal stem cells derived from human induced pluripotent stem cells modulate T-cell phenotypes in allergic rhinitis

**DOI:** 10.1111/j.1398-9995.2012.02875.x.

**Published:** 2012-08-01

**Authors:** Q L Fu, Y Y Chow, S J Sun, Q X Zeng, H B Li, J B Shi, Y Q Sun, W Wen, H F Tse, Q Lian, G Xu, Hans-Uwe Simon

**Affiliations:** 1 Otorhinolaryngology Hospital, The First Affiliated Hospital, Sun Yat-sen UniversityGuangzhou, Guangdong; 2 Cardiology Division, Department of Medicine and Research Centre of Heart, Brain, Hormone, and Healthy Aging, Li Ka Shing Faculty of Medicine, The University of Hong KongHong Kong; 3 Eye Institute, Li Ka Shing Faculty of Medicine, The University of Hong KongHong Kong, China

**Keywords:** allergic rhinitis, immunomodulation, induced pluripotent stem cells, mesenchymal stem cells, T cell

## Abstract

**Background:**

Human induced pluripotent stem cells (iPSCs) possess remarkable self-renewal capacity and the potential to differentiate into novel cell types, such as mesenchymal stem cells (MSCs). iPSC-MSCs have been shown to enhance tissue regeneration and attenuate tissue ischaemia; however, their contribution to the immune regulation of Th2-skewed allergic rhinitis (AR) and asthma remains unclear.

**Objective:**

This study compared the immunomodulatory effects of iPSC-MSCs and bone marrow-derived MSCs (BM-MSCs) on lymphocyte proliferation, T-cell phenotypes and cytokine production in peripheral blood mononuclear cells (PBMCs) in patients with AR, and investigated the possible molecular mechanisms underlying the immunomodulatory properties of iPSC-MSCs.

**Methods:**

In co-cultures of PBMCs with iPSC-MSCs or BM-MSCs, lymphocyte proliferation was evaluated using 3H-thymidine (3H-TdR) uptake, carboxyfluorescein diacetate, succinimidyl ester (CFDA-SE) assays; the regulatory T-cell (Treg) phenotype was determined by flow cytometry, and cytokine levels were measured using an enzyme-linked immunosorbent assay. The immunomodulatory properties of both MSCs were further evaluated using NS398 and transwell experiments.

**Results:**

Similar to BM-MSCs, we determined that iPSC-MSCs significantly inhibit lymphocyte proliferation and promote Treg response in PBMCs (*P* < 0.05). Accordingly, the cytokine milieu (IFN-γ, IL-4, IL-5, IL-10 and IL-13) in the supernatants of PBMCs changed significantly (*P* < 0.05). The immunomodulatory properties of iPSC-MSCs and BM-MSCs were associated with prostaglandin E2 (PGE2) production and cell–cell contact.

**Conclusions:**

These data demonstrate that iPSC-MSCs are capable of modulating T-cell phenotypes towards Th2 suppression through inducing Treg expansion, suggesting that iPSC-MSCs can be used as an alternative candidate to adult MSCs to treat allergic airway diseases.

Allergic rhinitis (AR) and asthma are characterised by Th2-skewed eosinophilic inflammation, mucus hypersecretion and airway hyperresponsiveness 1. The excessive activation of Th2 cells is thought to play a major role in initiating clinical features that include allergic sensitisation and driving airway hyperresponsiveness 2. Over the past few decades, there is growing evidence that the insufficient suppression of regulatory T cells (Treg) is responsible for the excessive Th2 response in allergic airway diseases 3. Therefore, a therapeutic strategy that reverses the established Th2 response by the adoptive transfer of regulatory T cells (Treg) 4–6, tolerogenic dendritic cells (DC) 7 and mesenchymal stem cells (MSCs) 8–12 may provide a potentially attractive approach for the management of allergic airway diseases.

Mesenchymal stem cells are ubiquitous multipotent cells that are abundant in adult bone marrow (BM) and adipose tissue 13,14. In addition to the potential for tissue repair, a growing body of evidence has demonstrated that BM-derived MSCs (BM-MSCs) display profound immunomodulatory capabilities, making them attractive candidates for the development of novel allogeneic cell-based therapeutic strategies in the treatment of a variety of inflammatory diseases 15–17. However, it has been reported that adult MSCs (e.g. BM-MSCs) exhibit several potential weaknesses, such as a limited proliferative capacity and the rapid loss of differentiation potential, which may affect their therapeutic efficacy 18–20. A recent breakthrough in the generation of human induced pluripotent stem cells (iPSCs) from adult somatic cells using reprogramming techniques 21 offers the possibility of generating a high yield of MSCs. However, the differentiation potential of human iPSCs into functional MSCs and their therapeutic efficacy in allergic airway diseases has not been well documented.

In a previous study, we successfully developed MSCs from human iPSCs (iPSC-MSCs) that are morphologically and functionally similar to BM-MSCs 22. Interestingly, we found that the transplantation of iPSC-MSCs significantly promotes vascular and muscular regeneration and attenuates severe hind-limb ischaemia in a mouse model. On the basis of the observation that iPSC-MSCs exhibit an immunomodulatory phenotype, we speculate that iPSC-MSCs may also function in the prevention of allergic airway diseases. To address this issue, we compared the immunomodulatory effects of iPSC-MSCs and BM-MSCs on peripheral blood mononuclear cells (PBMCs) in patients with AR and evaluated the possible molecular mechanisms underlying the immunomodulatory properties of these cells.

## Materials and methods

## Subjects

This study includes 22 patients with persistent allergic rhinitis (AR) and 34 healthy donors (the details are listed in Table S1). The diagnosis of persistent AR was in accordance with the criteria of the initiative from Allergic Rhinitis and its Impact on Asthma (ARIA) 1. All patients were positive for *Dermatophagoides pteronyssinus* (Der p1) (Pharmacia CAP system, Pharmacia Diagnostics, Uppsala, Sweden). The patients had not received antihistamines or intranasal steroid treatments for 1 week, or oral steroids for 3 months, prior to this study. The Ethics Committee of The First Affiliated Hospital, Sun Yat-sen University approved this study, and informed consent was obtained from all participants.

### Reagents

Anti-human CD3-PE and isotype-matched control were purchased from BD Biosciences (San Diego, CA, USA). The Treg cell kit containing anti-CD4-FITC, CD25-APC and Foxp3-PE antibodies was purchased from eBioscience (CA, USA). Enzyme-linked immunosorbent assay (ELISA) kits for human IFN-γ, IL-4, IL-5, IL-10, IL-13, transforming growth factor-β1 (TGF-β1) and prostaglandin E2 (PGE2) were purchased from R & D (Minneapolis, MN, USA).

### Generation of human iPSC-MSCs and identification of surface markers

Human iPSC-MSCs were prepared as we previously reported 22. The details are presented in Data S1.

### Lymphocyte proliferation assay

The details are presented in Data S1.

### Mixed lymphocyte reactions

The details are presented in Data S1.

### Flow cytometry analysis

The details are presented in Data S1.

### Enzyme-linked immunosorbent assay

The details are presented in Data S1.

### Prostaglandin inhibition

The details are presented in Data S1.

### Transwell experiments

The details are presented in Data S1.

### Statistical analysis

The data are expressed as the mean ± SEM. Statistical analysis was performed for the Treg cell assay between groups treated with and without MSCs using the paired Student's *t*-test. One-way analysis of variance (anova) followed by *post hoc* Tukey's multiple comparisons was used for multiple comparisons between different groups. Comparisons of two groups were performed using independent sample *t* tests. *P*-values < 0.05 were considered significant.

## Results

### iPSC-MSCs inhibit phytohaemagglutinin-stimulated PBMC proliferation

**Figure 1:**
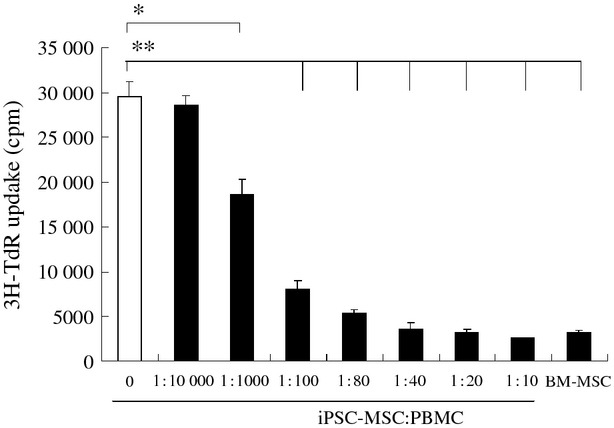
The immunosuppressive effect of iPSC-MSCs on PHA-stimulated lymphocyte proliferation. iPSC-MSCs significantly inhibited PHA-stimulated lymphocyte proliferation in a number-dependent manner, as determined by 3H-TdR uptake. BM-MSCs were used as a control. PBMCs from healthy volunteers (1 × 10^5^/well) were stimulated by PHA (5 μg/ml) in a 96-well plate for 3 days in the presence or absence of different numbers of iPSC-MSCs or BM-MSCs (1 × 10^4^/well) (*n* = 10). The data are expressed as the means ± SEM. *P < 0.05; **P < 0.01.

**Figure 2:**
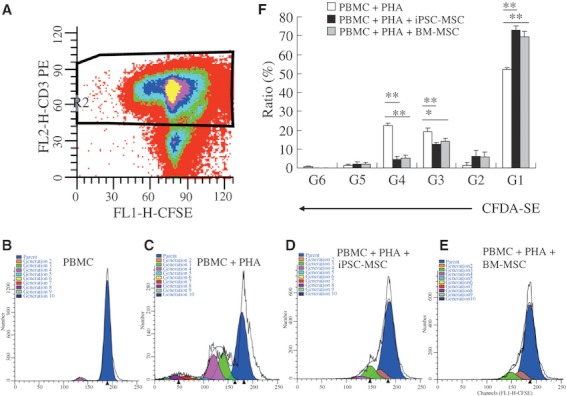
The immunosuppressive effect of iPSC-MSCs on PHA-stimulated T-cell proliferation. iPSC-MSCs suppressed T-cell proliferation in PHA-stimulated PBMCs, as determined by multicolour flow cytometric analysis. CFDA-SE-labelled PBMCs from healthy volunteers (5 × 10^5^ cells/well) were cultured in a 24-well plate for 3 days, and PBMCs were gated for flow cytometric analysis based on CD3^+^ staining (A). Representative results of T-cell proliferation without stimulation (B), PHA (5 μg/ml) stimulation (C), PHA stimulation in the presence of iPSC-MSCs (5 × 10^4^/well) (D) or BM-MSCs (E) are shown. Both iPSC-MSCs and BM-MSCs significantly increased the ratio of G1 T cells but decreased the ratio of G3 and G4 T cells in PHA-stimulated PBMCs (*n* = 10) (F). The data are expressed as the means ± SEM. G (generation) indicates the number of cell divisions. *P < 0.05; **P < 0.01.

To determine the immunomodulatory properties of iPSC-MSCs, we examined the effects of iPSC-MSCs on cell proliferation. As illustrated in Fig. 1, we found that in the 3H-TdR uptake experiment, iPSC-MSCs significantly inhibited PBMC proliferation at a ratio of 1 : 10 (10^4^ MSCs *vs* 10^5^ PBMCs), 1 : 20, 1 : 40, 1 : 80, 1 : 100 and 1 : 1000 (*P* < 0.05), suggesting iPSC-MSCs inhibited phytohaemagglutinin (PHA)-stimulated lymphocyte proliferation in a dose-dependent manner. Similarly, BM-MSCs significantly inhibited PHA-stimulated PBMC proliferation (10^4^ BM-MSCs *vs* 10^5^ PBMCs). Moreover, we examined the kinetics of the immunosuppression of iPSC-MSCs on T-cell proliferation using multicolour flow cytometric analysis. As illustrated in Fig. 2, we observed a significant accumulation of T cells in generation 1 (G1) (parent) and a significant decrease in T-cell accumulation in G3 and G4 after the addition of iPSC-MSCs or BM-MSCs (P < 0.05), suggesting that both iPSC-MSCs and BM-MSCs reduce the number of CD3^+^ T-cell divisions and inhibit cell proliferation. In contrast, no significant difference in the apoptosis of PBMCs, evaluated using annexin V and propidium iodide labelling, was observed (data not shown).

### iPSC-MSCs enhance Treg cell expansion in Der p1-stimulated PBMCs

**Figure 3:**
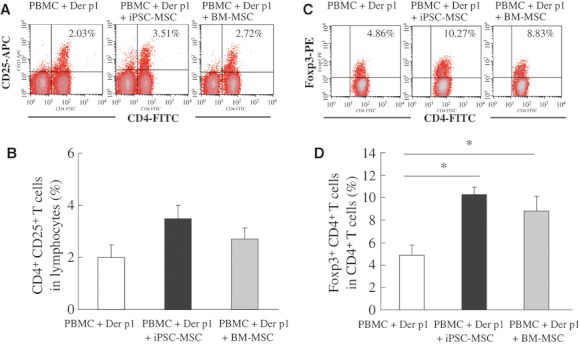
Induced pluripotent stem cells-MSCs enhanced the Treg frequency in Der p1-stimulated PBMCs, as determined by flow cytometry. PBMCs from allergic rhinitis (AR) patients (1 × 10^6^ cells/well) were cultured in a 24-well plate following Der p1 (10 μg/ml) stimulation in the presence or absence of iPSC-MSCs or BM-MSCs (5 × 10^4^/well) for 3 days. Representative flow cytometric results of CD4^+^CD25^+^ cells (A) and Foxp3^+^CD4^+^ cells (C) are shown. Both iPSC-MSCs and BM-MSCs significantly increased the frequency of Foxp3^+^CD4^+^ cells (D), but not CD4^+^CD25^+^ T cells (B), in the CD4^+^ subpopulation after Der p1 stimulation (*n* = 5 for each group). The data are expressed as the means ± SEM. *P < 0.05.

To investigate whether iPSC-MSCs modulate Treg cell expansion, we next examined the effects of iPSC-MSCs and BM-MSCs on Treg frequency in Der p1-stimulated PBMCs using flow cytometry. As illustrated in Fig. 3, we found no significant difference in CD4^+^CD25^+^ frequency in the CD4^+^ subpopulation after the treatments of both iPSC-MSCs and BM-MSCs. However, when we focused on Foxp3^+^CD4^+^ Treg cells, we found that both iPSC-MSCs and BM-MSCs significantly increased the ratio of Treg cells in the CD4^+^ subpopulation (*P* < 0.05), suggesting iPSC-MSCs exert a tolerogenic effect on the T-cell response. Interestingly, there was no significant difference in the Foxp3^+^CD4^+^ Treg frequency between the iPSC-MSCs and BM-MSCs groups.

### iPSC-MSCs regulate the cytokine production in Der p1-stimulated PBMCs

**Figure 4:**
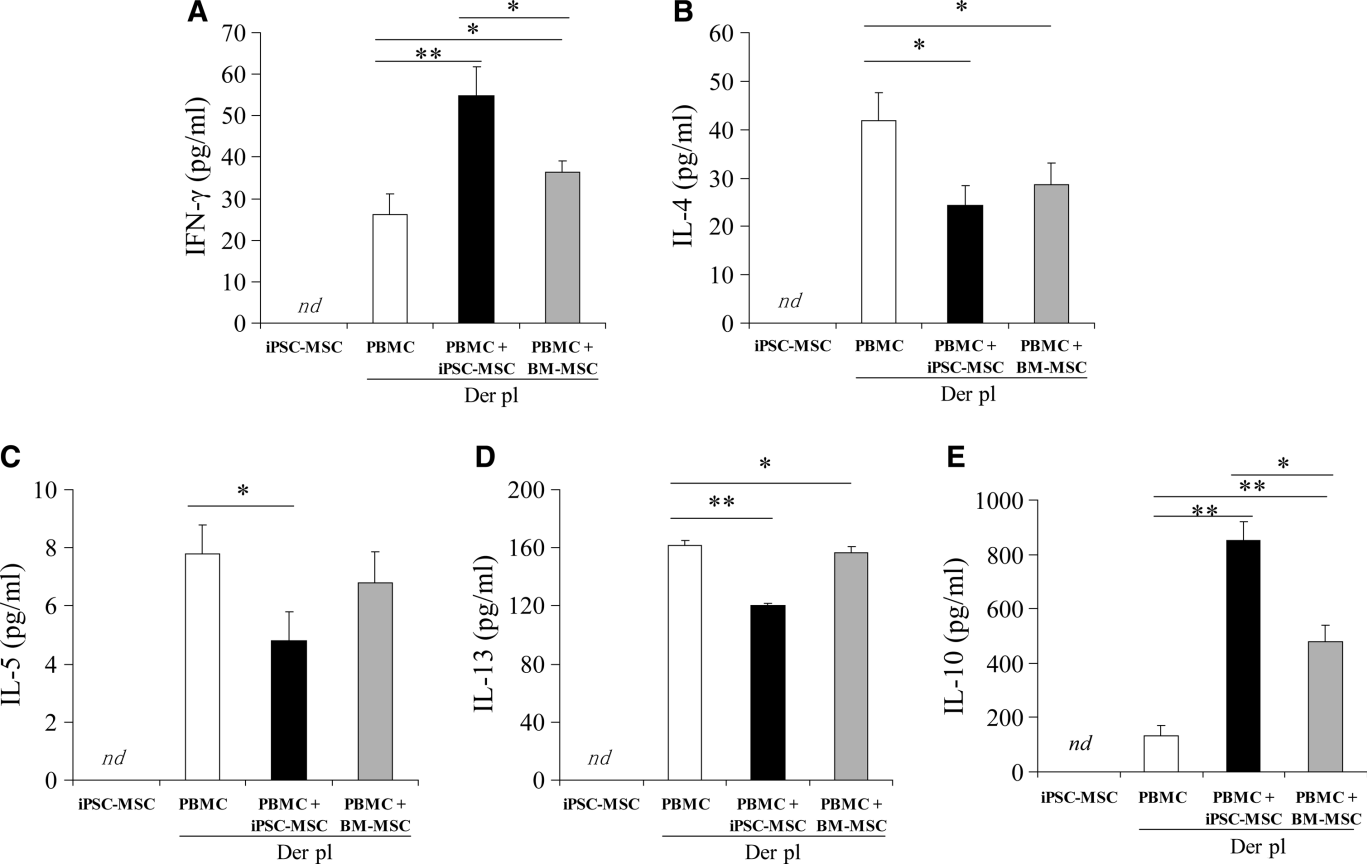
Induced pluripotent stem cells-MSCs-modulated cytokine levels in the supernatants of Der p1-stimulated PBMCs. PBMCs (1 × 10^6^ cells/well) collected from patients with AR were cultured in a 24-well plate following Der p1 (10 μg/ml) stimulation in the presence or absence of iPSC-MSCs or BM-MSCs (5 × 10^4^/well) for 3 days. The levels of IFN-γ, IL-4, IL-5, IL-13 and IL-10 in the supernatants of Der p1-stimulated PBMCs were determined using ELISA. iPSC-MSCs significantly increased the levels of IFN-γ (A) and IL-10 (B) but decreased the levels of IL-4 (C), IL-5 (D) and IL-13 (E) in the supernatants of Der p1-stimulated PBMCs. For each group, *n* = 11. The data are expressed as the means ± SEM. *P < 0.05; **P < 0.01. *nd*: no detectable.

To investigate further the immunomodulatory effects of iPSC-MSCs on the T-cell phenotype, we next examined the effects of iPSC-MSCs and BM-MSCs on cytokine production (IFN-γ representing the Th1 phenotype; IL-4, IL-5 and IL-13 representing the Th2 phenotype; and IL-10 representing the Treg phenotype) in the supernatants of Der p1-stimulated PBMCs using ELISA (Fig. 4). The addition of iPSC-MSCs or BM-MSCs significantly increased IL-10 levels in the iPSC-MSCs and BM-MSCs groups compared with the control group (*P* < 0.01), which was consistent with the increased Treg frequency observed in stimulated PBMCs. Interestingly, the IL-10 level in the iPSC-MSCs group was significantly higher than in the BM-MSCs groups (*P* < 0.05). On the other hand, the level of IFN-γ was significantly increased and the levels of IL-4, IL-5, IL-13 were significantly decreased in the iPSC-MSCs group compared with the control group (*P* < 0.05). Similarly, the level of IFN-γ was significantly increased and the level of IL-4 was significantly decreased in the BM-MSCs group compared with the control group (*P* < 0.05).

### The immunomodulatory properties of iPSC-MSCs are associated with cell contact and PGE2 production

**Figure 5:**
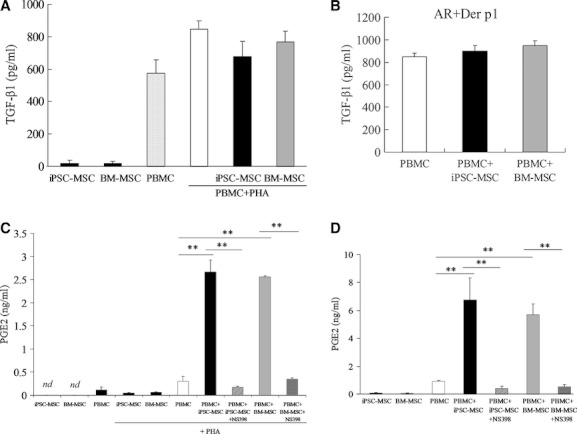
Induced pluripotent stem cells-MSCs or BM-MSCs modulated the levels of TGF-β1 and PGE2 in the supernatants of PHA or Der p1-stimulated PBMCs. (A, C) PBMCs from healthy donors (1 × 10^5^ cells/well) were cultured in a 96-well plate in the presence of iPSC-MSCs or BM-MSCs (1 × 10^4^ cells/well) for 3 days under the indicated conditions. (B, D) PBMCs from patients with AR (1 × 10^6^ cells/well) were cultured in a 24-well plate with 10 μg/ml Der p1 in the presence of iPSC-MSCs or BM-MSCs (5 × 10^4^ cells/well) for 3 days. Serum-free medium was used for the TGF-β1 experiments. The levels of TGF-β1 and PGE2 in the supernatants were determined using ELISA. A significant effect of both iPSC-MSCs and BM-MSCs on TGF-β1 level was not observed in the supernatants of PHA- or Der p1-stimulated PBMCs (A, B). However, both iPSC-MSCs and BM-MSCs significantly increased the PGE2 level in the supernatants of PHA- or Derp1-stimulated PBMCs, which was significantly inhibited by NS398 (C, D). For each group, *n* = 6. The data are expressed as the means ± SEM. **P < 0.01. *nd*: no detectable.

**Figure 6:**
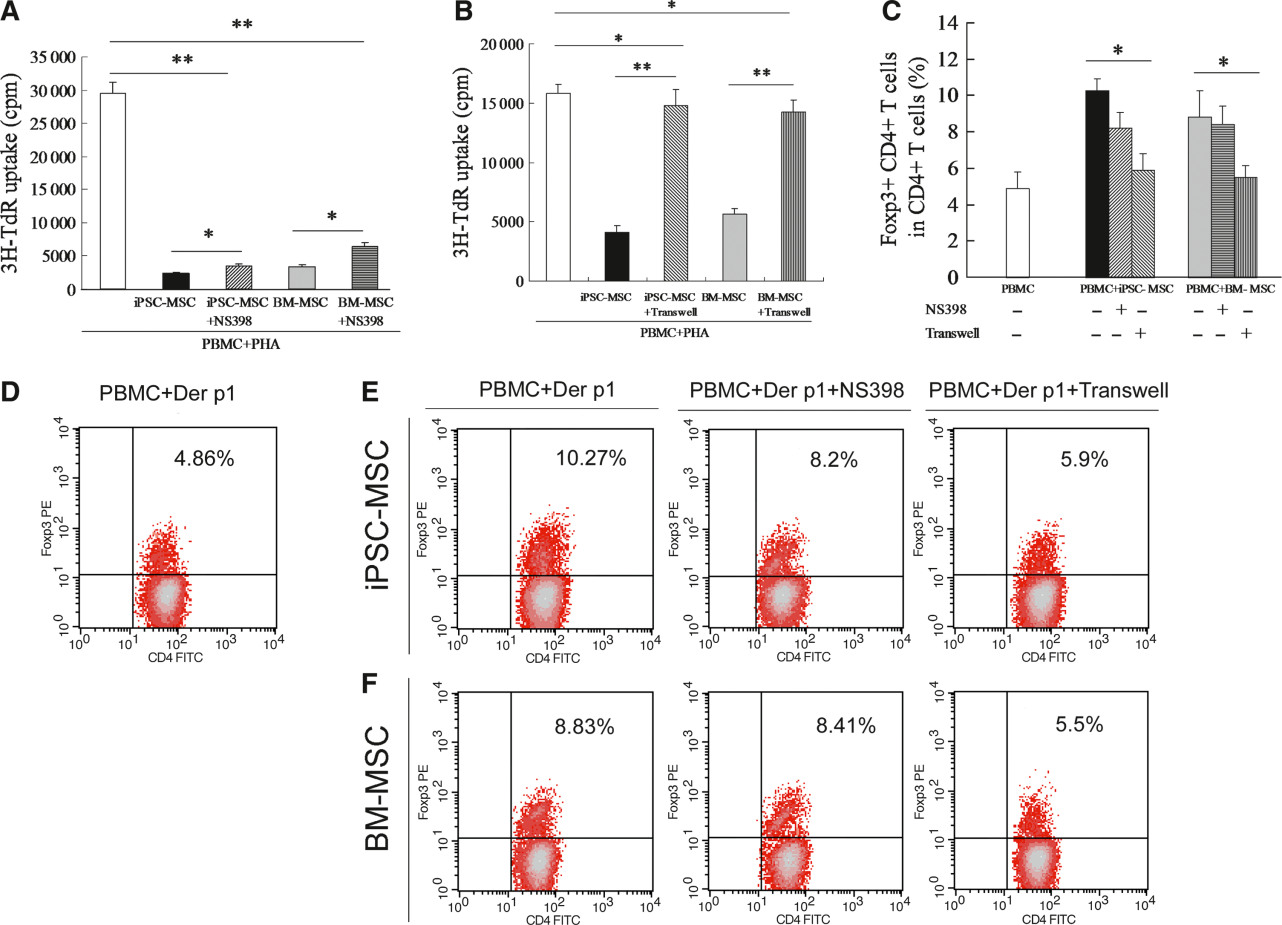
The immunomodulatory effects of iPSC-MSCs or BM-MSCs on lymphocyte proliferation and T regulatory cells' association with PGE2 production and cell–cell contact suppression. PBMCs from healthy volunteers were stimulated by PHA (5 μg/ml) for 3 days in the presence of iPSC-MSCs or BM-MSCs with NS398 (5 μM) or with transwell separation from MSCs. NS398 (A) and transwell separation (B) significantly reversed the immunomodulatory effect of iPSC-MSCs and BM-MSCs on PHA-stimulated lymphocyte proliferation, as suggested by 3H-TdR uptake (*n* = 10). PBMCs (1 × 10^6^ cells/well) collected from patients with AR were cultured in a 24-well plate with Der p1 (10 μg/ml) stimulation in the presence of iPSC-MSCs or BM-MSCs (5 × 10^4^ cells/well) with NS398 or without transwell separation from MSCs for 3 days. The transwell experiment significantly reversed the immunomodulatory effects of iPSC-MSC and BM-MSCs on the frequency of Foxp3^+^CD4^+^ cells in the CD4^+^ subpopulation of cultured PBMCs (C). Representative flow cytometric results in groups (D, Der p1-stimulated PBMCs; E, Der p1-stimulated PBMCs plus iPSC-MSCs; F, Der p1-stimulated PBMCs plus BM-MSCs) in PBMCs are shown. For each group, *n* = 5. The data are expressed as the means ± SEM. *P < 0.05, **P < 0.01. *nd*: no detectable.

To parallelly evaluate the molecular mechanisms underlying the immunomodulatory properties of iPSC-MSCs and BM-MSCs, we further examined the possible role of cell contact and PGE2 production in the modulation of Treg frequency and cytokine levels by MSCs. As illustrated in Fig. 5, both iPSC-MSCs and BM-MSCs had no significant effect on the level of TGF-β1 in the supernatants of PHA-stimulated PBMCs (from healthy volunteers) (Fig. 5A) or Der p1-stimulated PBMCs (from patients with AR) (Fig. 5B). However, the addition of iPSC-MSCs or BM-MSCs significantly increased the level of PGE2 in the supernatants of PHA- or Der p1-stimulated PBMCs, suggesting both types of MSCs increased PGE2 but not TGF-β1 production in stimulated PBMCs (*P* < 0.05). Interestingly, the level of PGE2 decreased significantly after the addition of NS398, a specific COX-2 inhibitor. Moreover, NS398 significantly reversed the immunomodulatory effect of iPSC-MSCs or BM-MSCs on PHA-stimulated lymphocyte proliferation, as suggested by the 3H-TdR uptake assay (Fig. 6A). In addition, similar to NS398, transwell separation significantly reversed the immunomodulatory effect of iPSC-MSCs and BM-MSCs on PHA-stimulated lymphocyte proliferation (Fig. 6B). Moreover, transwell separation significantly reversed the immunomodulatory effects of iPSC-MSC and BM-MSCs on the frequency of Foxp3^+^CD4^+^ T cells in the CD4^+^ subpopulation in Der p1-stimulated PBMCs (from patients with AR) (Fig. 6C–F). These findings suggest that both PGE2 and cell contact prevented the iPSC-MSCs and BM-MSCs from exerting immunomodulatory effects on lymphocyte proliferation and the Treg phenotype.

## Discussion

In this study, we took advantage of a short-term, *in vitro* culture system to provide preliminary evidence that similar to BM-MSCs, human iPSC-MSCs exert immunomodulatory effects on T-cell phenotypes in PBMCs from patients with AR. To our knowledge, this is the first study to compare the potential of iPSC-MSCs and BM-MSCs in the modulation of the allergic response, shedding light on the clinical use of iPSC-MSCs for the prevention of allergic airway diseases.

Over the last decade, MSCs have attracted significant interest because of their ability to develop into almost any cell type, which makes them attractive for regenerative medicine. In addition, the importance of MSCs derived from the BM and adipose tissues in the regulation of allergic airway diseases has been addressed. For example, Cho et al. reported that adipose tissue-derived MSCs significantly reduced allergic symptoms and inhibited eosinophilic inflammation in the nasal mucosa in an ovalbumin-induced AR mouse model 8. Kavanagh et al. reported that the systemic administration of allogenic BM-MSCs protected the airways from allergen-induced pathology by inducing Foxp3^+^CD4^+^ Treg cells 11. More recently, Kapoor et al. demonstrated that BM-MSCs suppress the proliferation of allergen-challenged PBMCs from asthmatic subjects through an *in vitro* stimulation system 12. Although MSCs have not been used in the clinic, these findings demonstrate the potential beneficial role of these cells for the management of allergic airway diseases.

In our previous study 22, we successfully isolated three monoclonal, karyotypically stable and functional iPSC-MSCs using a combination of CD24 and CD105 sorting. We demonstrated that these cells did not express pluripotent-associated markers but displayed MSC surface antigens and differentiated into adipocytes, osteocytes and chondrocytes. Moreover, transplanting iPSC-MSCs into mice significantly attenuated severe hind-limb ischaemia, and promoted vascular and muscle regeneration. Notably, we determined that the beneficial effects of iPSC-MSCs on limb ischaemia were superior to those of adult BM-MSCs, suggesting that iPSC-MSCs are important tools for tissue repair because of their multipotency properties. However, whether iPSC-MSCs possess immunosuppressive properties and function in the modulation of allergic airway diseases is still unclear. To identify the immunomodulatory properties of iPSC-MSCs, we first examined the expression of MHC and costimulatory molecules on iPSC-MSCs in this study. In agreement with previous reports about iPSC-MSCs 23 and BM-MSCs 24,25, we found that similar to human BM-MSCs, human iPSC-MSCs express HLA class I (ABC), low levels of HLA class II (DR), but not CD80, CD86 or CD40 (data not shown). This observation suggests the similarity between the immunosuppressive phenotypes of iPSC-MSCs and BM-MSCs *in vitro*. Although iPSCs arising from reprogrammed adult somatic cells represent an interesting alternative to embryonic stem cells, the immunosuppressive activity of iPSC-MSCs has still to be explored and compared with BM-MSCs. For this reason, we examined further the immunomodulatory effects of iPSC-MSCs and BM-MSCs on cell proliferation in PHA-stimulated PBMCs. As expected, iPSC-MSCs significantly inhibited PHA-stimulated lymphocyte proliferation in a dose-dependent manner. Using multicolour flow cytometric analysis, we determined that there was a significant accumulation of T cells in G1, which was followed by a significant decrease in T-cell accumulation in G3 and G4 after the addition of iPSC-MSCs or BM-MSCs. Our data suggested that both iPSC-MSCs and BM-MSCs reduced the number of CD3^+^ T-cell divisions and inhibited cell proliferation. Considering that the immunosuppressive function of iPS-MSCs is maintained during multiple passages without any senescence, which is in contrast to BM-MSCs (10 passages *vs* 3 passages for the BM-MSCs), it is plausible that compared to BM-MSCs, iPSC-MSCs may offer a long-lasting, more efficient opportunity to inhibit cell proliferation in PBMCs.

Mesenchymal stem cells are not globally suppressive and MSC-mediated immune modulation has generally been linked to increases in Treg cell numbers and related cytokines 26. However, the immunosuppressive effect of iPSC-MSCs on the PBMCs from patients with AR is still unclear. In this study, we compared the effects of iPSC-MSCs and BM-MSCs on T-cell phenotypes in PBMCs from patients with AR. As expected, the levels of Th2 cytokines (IL-4, IL-5, and IL-13) were significantly decreased after the addition of iPSC-MSCs or BM-MSCs, whereas IL-10 levels were significantly increased. We have consistently found that both iPSC-MSCs and BM-MSCs significantly increased the ratio of Treg cells in the CD4^+^ subpopulation. Interestingly, a significant difference in Foxp3^+^CD4^+^ Treg frequency between iPSC-MSCs and BM-MSCs groups was not observed. Our findings, and those from other reports, provide evidence that Treg expansion plays a central role in the immunomodulatory properties of both iPSC-MSCs and BM-MSCs.

The mechanisms underlying iPSC-MSCs-mediated Treg expansion and Th2 suppression in PBMCs are unclear. In previous studies, apart from the importance of DC modulation 12, the exact mechanisms underlying Treg induction after the administration of BM-MSCs in allergic airway diseases have not been identified. It is recognised that adult MSCs exert immunomodulatory effects on immune cells through cell–cell contact and soluble inhibitory factors 26. Although the published literature overwhelmingly demonstrates evidence of Treg-related suppression through the induction of the inhibitory cytokine TGF-β1 27, in this study, we did not observe a significant change in TGF-β1 levels in the supernatants of cultured PBMCs. However, we observed that iPSC-MSCs significantly increased PGE2 levels in the supernatants of PHA- or Derp1-stimulated PBMCs (from healthy controls or patients with AR, respectively), suggesting that PGE2 might be the cytokine responsible for lymphocyte proliferation and Treg expansion in PBMCs. This hypothesis was further substantiated using NS398, a specific COX-2 inhibitor for lymphocyte proliferation. Moreover, transwell separation significantly reversed the immunomodulatory effect of iPSC-MSCs and BM-MSCs on lymphocyte proliferation as well as Treg induction, providing evidence that cell contact, in addition to cytokine production, was the key mechanism utilised by iPSC-MSCs in this short-term, *in vitro* culture system.

Mesenchymal stem cells derived from the BM are used in most preclinical and clinical studies. But the limited capacity to proliferate and the quick loss of their differentiation potential limit their therapeutic efficacy to some extent. iPSCs can generate high yields of MSCs, and patient-specific iPSC-generated MSCs can be used for autologous transplantation without the need for immunosuppression. This pilot study parallelly identified the ability of iPSC-MSCs and BM-MSCs to immunomodulate T-cell phenotypes in cultured PBMCs from patients with AR. It suggests that iPSC-MSCs can be used as an alternative candidate to adult MSCs to treat allergic airway diseases. However, it is noteworthy that iPSC-MSCs present several challenges. For example, the low efficiency of iPSC generation and the viral integration of transgenes cannot be ignored. Moreover, for now, the epigenetic memory, immune rejection and teratoma formation will also limit the preclinical and clinical use of cells such as these 28. Alternatively, the recent development of methodologies for inducing iPSCs will render iPSC-MSCs an attractive candidate for biotherapies.

We acknowledge that this study contains a number of flaws. For example, the short-term culture system does not reflect the precise action of iPSC-MSCs, and mechanisms other than PGE2 production and cell–cell contact cannot be excluded. Moreover, the validation of the *in vitro* iPSC-MSCs data on T-cell modulation cannot be overestimated. Therefore, it will be of value to perform further *in vivo* studies to clarify whether iPSC-MSCs efficiently regulate T cells and protect against airway inflammation in an AR mouse model.

In conclusion, we have provided preliminary evidence of the immunomodulatory properties of iPSC-MSCs and BM-MSCs for the modulation of T-cell phenotypes in AR. Because iPSC-MSCs may provide an immeasurable source of autologous cells that can be prepared from individual patients, this comparative study has identified iPSC-MSCs as an attractive candidate for the management of allergic inflammation in a patient-specific, cost-effective, and batch-to-batch consistent manner.

## Supporting Information

**Table S1.** Numbers of the patients and healthy donors used in this study.

**Data S1.** Generation of human iPSC-MSCs and identification of surface markers.
