# Supplementary material for: Mesenchymal stem cells derived from human induced pluripotent stem cells modulate T-cell phenotypes in allergic rhinitis
Source: Allergy. 2012 Aug 1;67(10):1215–22. doi: 10.1111/j.1398-9995.2012.02875.x. (PMC3555482; doi:10.1111/j.1398-9995.2012.02875.x.)
Supplement: Supplementary file 1 [file all0067-1215-SD1.doc]

Table S1 Numbers of the patients and healthy donors used in this study

|  | Proliferation analysis | | | | | | | Mixed lymphocyte reaction | | |
| --- | --- | --- | --- | --- | --- | --- | --- | --- | --- | --- |
|  |  |  | |  | | Supernatant | | Supernatant | |  |
|  | 3H-TdR | | CFDA-SE | | 3H-TdR (Transwell) | TGF-β1 | PGE2 | Cytokines, PGE2 | TGF-β1 | Treg |
| Healthy donors | 10* | | 6 | | 6 | 6 | 6 |  |  |  |
| AR patients |  | |  | |  |  |  | 11 | 6 | 5^#^ |

For the proliferation analysis, the PBMCs derived from healthy donors were stimulated by phytohaemagglutinin (PHA) in the absence or presence of iPSC-MSC or BM-MSCs. MLR (mixed lymphocyte reaction): the PBMCs derived from allergic rhinitis (AR) patients stimulated by Der p1 were cultured for 3 days in the absence or presence of iPSC-MSC or BM-MSCs. *: the PBMCs were also used to assay the inhibition of prostaglandin E2 production (6 samples per group). ^#^: the PBMCs were also used to assay the inhibition of prostaglandin E2 production and transwell experiments. CFDA-SE: carboxyfluorescein diacetate, succinimidyl ester; 3H-TdR: 3H-thymidine; Treg: regulatory T cells;
