# Supplementary material for: Mesenchymal stem cells derived from human induced pluripotent stem cells modulate T-cell phenotypes in allergic rhinitis
Source: Allergy. 2012 Aug 1;67(10):1215–22. doi: 10.1111/j.1398-9995.2012.02875.x. (PMC3555482; doi:10.1111/j.1398-9995.2012.02875.x.)
Supplement: Supplementary file 2 [file all0067-1215-SD2.doc]

**Supporting Information**

**Generation of human iPSC-MSCs and identification of surface markers**

Briefly, iPSC (iMR90)-4 (WiCell Research Institute，Madison, WI, USA) and iPSC (iMR90)-5 (ATCC catalog No. CCL-186) generated from IMR90 fibroblast cells were used for MSC differentiation. A confluent 6-cm plate of iPSCs was trypsinized for 3 minutes at 37°C and placed on a gelatinized 10-cm dish containing knockout Dulbecco modified Eagle’s medium (DMEM, GIBCO, Invitrogen Corporation, CA, USA) supplemented with 10% serum replacement medium (GIBCO), basic fibroblast growth factor (bFGF, 10 ng/ml, GIBCO), platelet-derived growth factor AB (10 ng/ml, Peprotech, Rocky Hill, NJ, USA), and epidermal growth factor (10 ng/ml, Peprotech,) for enrichment of MSC outgrowth. After 1 week, differentiating iPSCs were harvested and incubated with CD24-PE and CD105-FITC (BD PharMingen, CA, USA). The CD24^-^CD105^+^ cells from the differentiating iPSCs, sorted using a fluorescence-activated cell sorting (FACS) system, were seeded in a 6-well plate beginning with 10 000 cells per well under knockout DMEM plus 10% FCS (GIBCO), bFGF (5 ng/ml), platelet-derived growth factor AB (10 ng/ml), and epidermal growth factor (10 ng/ml). The CD24^-^CD105^+^ cells were diluted with 0.5 cell per well in a 96-well plate after they were confluent. When the clones derived from a single cell were grown up to 60% to 70% of confluence, the cells from each well were reseeded into 1 well of 6-well plates and serially reseeded thereafter in 25-,75-, and 175-cm^2^ flasks at a density of 1×10^4^/ml. When cells were confluent in 175-cm^2^ tissue culture flasks, they were set as passage 1 and frozen down as cell stocks; 8 clone lines were achieved in this manner. Clones of iPSC-MSC9 [from iPSC(iMR90)-4] and iPSC-MSC10 [from iPSC(iMR90)-5] were selected for this study after a series of repassages (passage 3-9). Characterised adult human BM-MSCs were purchased from Cambrex BioScience, Rockland, ME, USA (catalogue no. PT-2501).

**Lymphocyte proliferation assay**

Peripheral blood mononuclear cells (PBMCs) were isolated using Ficoll-Plaque (Amersham Biosciences, NJ, USA). The aliquoted PBMCs were stored at liquid nitrogen until used. The effects of iPSC-MSCs and BM-MSCs on lymphocyte proliferation were examined using 3H-thymidine (3H-TdR) uptake and carboxyfluorescein diacetate, succinimidyl ester（CFDA-SE）labelling. For the analysis of 3H-TdR uptake, different numbers of human iPSC-MSCs (10, 1 × 10^2^, 1 × 10^3^, 1.25 × 10^3^, 2.5 × 10^3^, 5 × 10^3^, and 1 × 10^4^ cells/well) and human BM-MSCs (10^4^ cells/well) were plated onto 96-well plates and were allowed to adhere to the plate for 1-2 hours (*n* = 10). Human PBMCs (1 × 10^5^ cells/well) were stimulated by phytohaemagglutinin (PHA, 5 µg/ml, Sigma, MO, USA) for 3 days and 3H-TdR（1 µ Ci [0.037 MBq], ShangHai Institute of Applied Physics, ShangHai, China) was added 16 hours before the culture completion. Next, the cells were harvested onto glass microfibre filters and the incorporated radioactivity was measured in a 1450 Microbeta TriLux apparatus (Tri-Carb 2900RT, Perkin Elmer, Boston, MA). The lymphocyte proliferation was expressed as the incorporated radioactivity in counts per minute (cpm).

For the analysis of cell division using CFDA-SE labelling, PBMCs (5 × 10^5^ cells/well) stained with CFDA-SE (Sigma, MO, USA) were stimulated with PHA (5 µg/ml) in 24-well plates for 3 days (*n* = 6) in the presence of iPSC-MSCs or BM-MSCs (5 × 10^4^ cells/well). Next, gated CD3^+^ T-cells in PBMCs were analysed using a FACSCalibur cytofluorimeter and CellQuest software (BD Biosciences, NJ, USA). The number of cell divisions was determined by sequential halving of the CFDA-SE fluorescence and six individual peaks (G1-G6) and their corresponding regions were evaluated.

**MLR**

PBMCs (1 × 10^6^ cells/well) from AR patients were stimulated with 10 µg/ml Der p1 (Indoor Biotechnologies, United Kingdom) in 24-well plates for 3 days in the presence of iPSC-MSCs or BM-MSCs (5 × 10^4^ cells/well). Next, PBMCs were collected and Treg cells (*n* = 5) were analysed using flow cytometry. The levels of IL-4, IL-5, IL-10, IL-13, IFN-γ and PGE2 in the supernatants were examined using ELISA (*n* = 11).

**Flow cytometry analysis**

Flow cytometry analysis was performed to identify Treg cells using a BD FACSCalibur flow cytometer (BD Biosciences, NJ, USA) in accordance with the manufacturer’s instructions. PBMCs were surface-stained with CD4 and CD25 and were stained intracellularly with Foxp3. A minimum of 20,000 events were collected in each analysis. Isotype-matched immunoglobulin was used as negative control for each assay.

**ELISA**

The concentrations of human IL-4, IL-5, IL-10, IL-13, IFN-γ and PGE2 in the supernatants of PBMCs were examined using commercially available ELISA kits in accordance with the manufacturer’s instructions. The detection limits were as follows: 10 pg/mL for IL-4; 0.29 pg/mL for IL-5; 3.9 pg/mL for IL-10; 32 pg/mL for IL-13; 7.8 pg/mL for IFN-γ and 30.9 pg/mL for PGE2. For convenient analysis, all values below the detection limits were set at 0.

**TGF-β1 assay**

For the TGF-β1 assay, the PBMCs (1 × 10^6^ cells/well) from AR patients were stimulated with 10 µg/ml of Der p1 in a 24-well plate in the presence of iPSC-MSCs or BM-MSCs (5 × 10^4^ cells/well) for 3 days in serum-free medium (*n* = 6) (26). In the proliferation experiment, the PBMCs from healthy volunteers were treated with PHA and iPSC-MSCs or BM-MSCs in a 96-well plate for 3 days in serum-free medium (*n* = 6). The level of TGF-β1 in the supernatant was determined using ELISA in accordance with the manufacturer’s instructions.

**Prostaglandin inhibition**

PGE2 production inhibitor NS-398 (5 µM; Cayman Chemicals, Ann Arbor, MI) was used to inhibit PGE2 production. We examined the co-culture effect of NS-398 with iPSC-MSCs or BM-MSCs on lymphocyte proliferation by PHA on day 3. NS-398 was also used to examine the effect of PGE2 on regulating immunomodulation of iPSC-MSCs or BM-MSCs on Treg phenotype for PBMCs from AR patients in the treatment of DerP1. The number is 6 for each group.

**Transwell experiments**

For the transwell experiments, 24-well transwell plates with a 0.4 μm pore membrane (Costar, Corning, NY) were used to separate the PBMCs from the iPSC-MSCs or BM-MSCs. iPSC-MSCs or BM-MSCs were plated into the lower chamber at 5 × 10^4^ cells/well, and PHA-stimulated human PBMCs (5 × 10^5^ cells/well) were cultured in the upper chamber of the transwell insert. After 3 days of co-culture, the PBMCs were harvested and placed in a 96-well plate at a concentration of 1 × 10^5^ cells/well (n=6), were pulsed with 3H-TdR continuously for 16 hours and the cpm value was measured to determine the role of cell-cell contact in the immunomodulatory effect of iPSC-MSCs or BM-MSCs. Similarly, PBMCs from AR patients were stimulated by Der p1 in transwell chamber plates for 3 days in the presence or absence of iPSC-MSCs or BM-MSCs (*n* = 6). Next, the cells were harvested for Treg analysis using flow cytometry as described above.
